# Finger Fasciotomy Due to Compartment Syndrome Caused by Inoculation of Hydraulic Oil by a Pressure Machine

**DOI:** 10.7759/cureus.111152

**Published:** 2026-06-19

**Authors:** Manuel Cerdeiros, Olga Tkachuk, André Ramos, André Vasques, Francisco Brito

**Affiliations:** 1 Department of Orthopedics and Trauma, Unidade Local de Saúde do Baixo Alentejo, Beja, PRT

**Keywords:** compartment syndrome, finger fasciotomy, intracompartmental pressure, necrosis, pinprick

## Abstract

Isolated finger compartment syndrome is a rare disorder and is sparsely recorded in the literature. Fascial compartment pressure builds up, reducing perfusion to the finger and potentially leading to irremediable injury. We present a case of a man with severe pain who arrived at the hospital nine hours after he wounded his right ring finger in a high-pressure machine. The fingertip was pale and swollen, with a small lesion on the volar side. Capillary refill test and the pinprick test were abnormal, and radiographs showed no fracture. Finger fasciotomy was performed immediately through a radial coronal incision. The wound was left open, and on the third day after surgery, the finger showed early and clear signs of improvement. A rapid diagnosis and an urgent decompression are extremely crucial in the treatment and prognosis of the patient.

## Introduction

Acute compartment syndrome is a widely recognized orthopedic emergency, but when it strikes the highly confined space of a single digit, it becomes a rare, notoriously deceptive, and potentially devastating clinical entity [1]. It develops as a result of reduced intracompartmental space or increased intracompartmental fluid volume because the enclosing fascia is intrinsically nonyielding and will limit expansion [2]. As the compartment pressure rises, hemodynamics become affected, and a decrease in tissue oxygenation is expected. This will lead to acute pain and swelling, tissue necrosis, and functional morbidity [3,4].

High-pressure injection injuries to the hand are uncommon but represent true orthopedic emergencies. They typically occur in industrial settings when equipment such as paint, oil, or grease guns accidentally discharge into the digit. These injuries are notoriously deceptive; they often present with a benign-appearing, pinpoint entry wound that severely masks extensive internal damage. The injected material spreads rapidly along fascial planes, causing immediate mechanical disruption of tissue, followed by a profound chemical inflammatory response. The combination of the injected foreign volume and the intense ensuing edema within the tight, noncompliant boundaries of the finger rapidly elevates intracompartmental pressure. This cascade creates a perfect storm for the development of acute digital compartment syndrome, making prompt clinical recognition vital to prevent extensive tissue necrosis and subsequent amputation. Finger fasciotomy, as an emergent procedure, can decrease intracompartmental pressure, preventing disastrous consequences.

We present this case to highlight the uniqueness of an isolated finger compartment syndrome caused by high-pressure oil injection. Furthermore, this report emphasizes that even with a significantly delayed presentation of nine hours and in the absence of intracompartmental pressure monitors, relying on a prompt clinical diagnosis and urgent fasciotomy can successfully salvage the digit, prevent amputation, and preserve full functional range of motion. Because this is a very rare incident in which standardized treatment guidelines are still lacking, documenting such successful outcomes is of utmost importance to the medical literature.

## Case presentation

A 28-year-old man with no relevant personal or family history had a work accident, in which he was injected with hydraulic oil into his right ring finger by a high-pressure coal mine machine. Nine hours later, he presented to the Emergency Department with exorbitant pain of the proximal and middle phalanges and hypoesthesia of the distal third of the finger.

**Figure 1:**
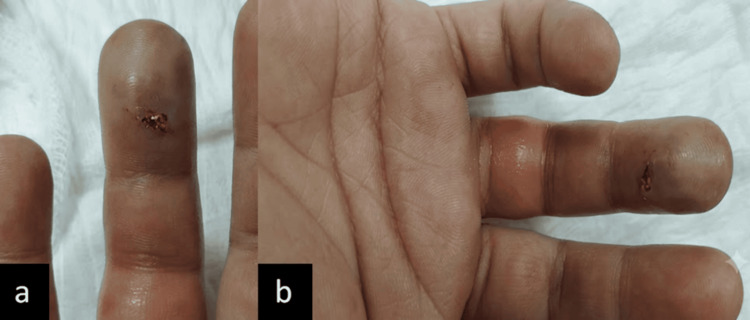
(a) Photograph of the patient's right ring finger injected with hydraulic oil by a high-pressure coal mine machine. (b) Photograph showing a puncture wound in the ring finger, associated with edema and pallor

The volar zone 1 of the finger showed a 6-mm puncture wound associated with clear macroscopic signs of ischemia. We could see exuberant edema and pallor of the entire finger (Figure 1).

**Figure 2:**
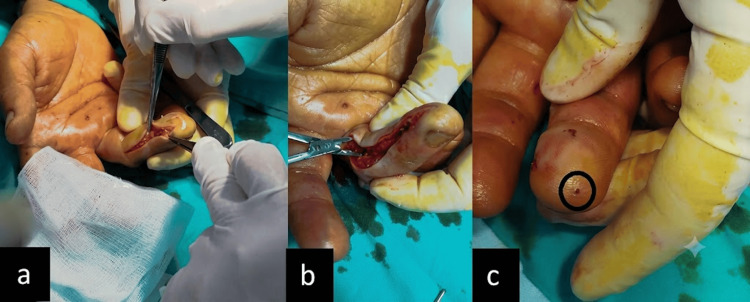
(a,b) Photographs showing a finger fasciotomy by unilateral coronal radial incision. (c) Pinprick test positive after fasciotomy

Anteroposterior and lateral radiographs were performed, which did not show any signs of fracture. A clinical diagnosis of acute compartment syndrome of the finger was immediately established. Emergent fasciotomy through a unilateral coronal incision was performed immediately by a radial approach, and the capillary refill test and the pinprick test were immediately normal. Then, the wound was left open for secondary intention closure (Figure 2).

**Figure 3:**
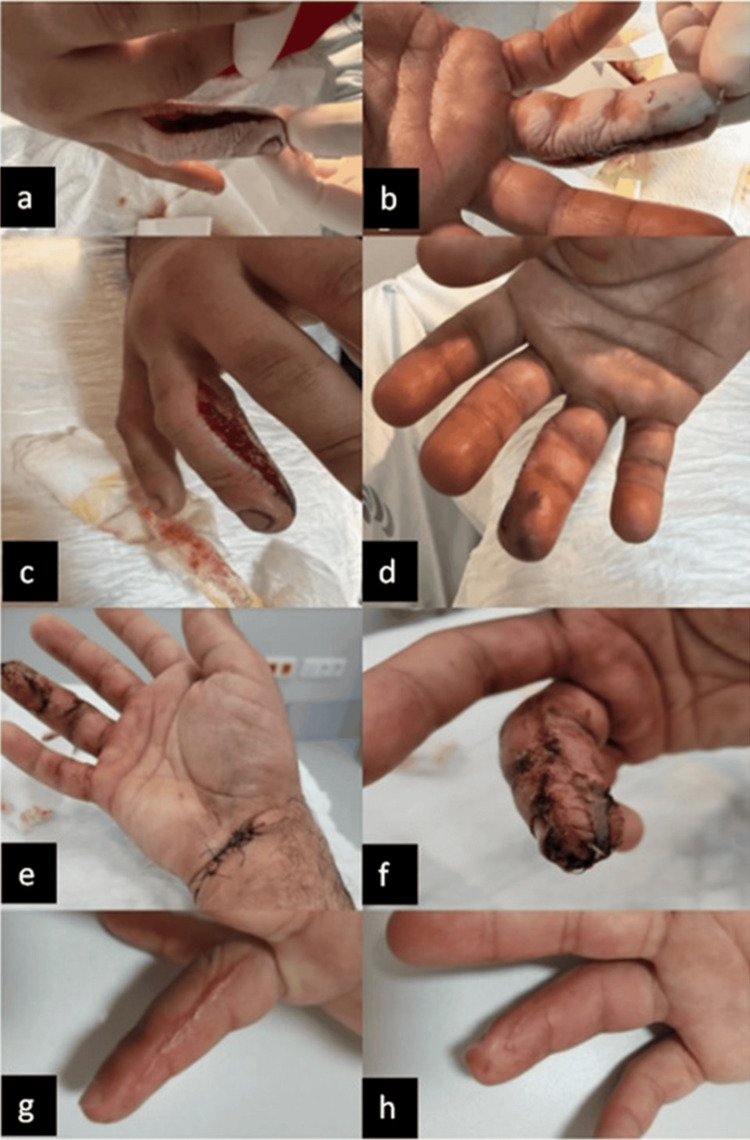
(a,b) One day postoperative, where the entire finger was pale and numb, with signs of skin necrosis. (c,d) Third day postoperative, where the color of the finger had returned to normal and a small volar area of necrosis on the fingertip started to appear. (e,f) Three weeks postoperative, where a transverse wrist crease flap was harvested and transferred. (g,h) One year postoperative, where the patient has a normal finger with full range of motion and sensation

On the first day after the procedure, the entire finger was pale and numb, with signs of poor vascularization. To optimize the outcome, postoperative measures included limb elevation, prophylactic antibiotics (amoxicillin/clavulanic acid), and daily dressing changes. Furthermore, a rehabilitation protocol consisting of early active and passive mobilization was initiated to prevent joint stiffness. On the third day after surgery, the color of the finger had returned to normal, and the sensation was partially restored. We could still see a little edema of the entire finger, a small volar area of necrosis on the fingertip, and the appearance of granulation tissue in the incision area. After three weeks, the patient had a necrotic volar zone 1 of the digitus annularis, which was debrided, and a transverse wrist crease flap was harvested and transferred. The wound healed completely. At the 12-month follow-up, the patient was fully asymptomatic, demonstrating complete active flexion and extension of all finger joints and intact tactile sensation across the entire digit (Figure 3).

## Discussion

Compartment syndrome is a surgical emergency that can compromise limb perfusion and ultimately lead to tissue necrosis. Nowadays, we know that many factors can increase the interstitial tissue pressure and consequently lead to reduced perfusion and local tissue hypoxia [5]. If this event develops over time without surgical intervention, it can lead to necrosis and an increase in morbidity and mortality [6].

While general compartment syndrome and its causes are widely documented, available literature rarely explores acute compartment syndrome involving an isolated finger [7]. It is a very rare incident, and correct treatment guidelines and consensus are still lacking.

Regarding the specific mechanism of injury, high-pressure injection injuries to the hand are devastating events with a notoriously high risk of amputation. Materials such as hydraulic oil spread rapidly along the fascial planes, causing mechanical distension, severe chemical inflammation, and vascular compression. This intense cascade quickly leads to a dramatic rise in intracompartmental pressure, precipitating an acute compartment syndrome. While specific guidelines for isolated digital compartment syndrome are scarce, the literature is unanimous regarding high-pressure injections: prompt recognition, urgent surgical decompression, and meticulous debridement are the gold standard to preserve tissue viability and maximize functional outcomes [8].

While the classic "5 P's" (pain, pallor, paresthesia, pulselessness, and paralysis) are historically cited, relying on them is a clinical pitfall, as signs like pulselessness are late indicators of irreversible ischemia. Instead, the rapid diagnosis of acute compartment syndrome relies almost exclusively on clinical vigilance regarding pain, specifically, severe pain out of proportion to the apparent injury and pain exacerbated by passive stretching [9]. However, with a diagnosis comes a treatment, and in the available literature, Codding et al. reported that decompression should be performed when tissue pressure is within 30 mmHg of the patient's diastolic blood pressure [10]. In the absence of intracompartmental pressure monitors in our hospital and because the patient presented to the emergency department with such clear clinical findings, an emergent finger fasciotomy through a unilateral radial incision was done immediately to prevent major consequences.

To fully understand the rapid onset of digital compartment syndrome, one must consider the unique anatomy of the finger pulp. In the finger, the two neurovascular bundles on the radial and ulnar sides are restrained by Cleland’s and Grayson’s ligaments [11]. The volar aspect of the digit is compartmentalized by dense fibrous septa that anchor the dermis to the underlying periosteum and tendon sheaths. Because these spaces are enclosed and noncompliant, the introduction of foreign fluid results in a dramatic rise in intracompartmental pressure. This pressure rapidly constricts the neurovascular bundles within these tight spaces, obstructing blood flow and precipitating ischemia. Consequently, effective surgical treatment requires not just a superficial incision, but the meticulous division of these fibrous septa to fully decompress the digital compartments and restore tissue perfusion [12]. It is proven that irreversible tissue damage remains if the time until treatment exceeds eight hours [13]. Our present case directly attests to this principle, as the patient's delayed presentation to the emergency department resulted in localized skin necrosis, emphasizing the critical importance of early surgical intervention.

In this case, the patient presented to the Emergency Department nine hours after the incident, which inevitably led to necrosis of a small volar region in the distal finger. Nevertheless, proper digital decompression was done, which prevented more nefarious consequences to the patient, like amputation of the finger.

## Conclusions

This is a rare case of isolated finger compartment syndrome, whose cause was a penetrating wound from a high-pressure machine. This case underscores the crucial lesson that clinicians must maintain a high index of suspicion, as the benign appearance of a pinpoint puncture wound often severely masks extensive internal tissue damage. Diagnosis must be made clinically driven by severe pain out of proportion to the apparent injury, rather than waiting for late ischemic signs or relying on intracompartmental pressure monitors. Furthermore, this case perfectly illustrates the concept that "time is tissue." Due to the delayed presentation for decompression surgery, a secondary reconstructive procedure with a skin flap was required. Ultimately, early recognition, immediate fasciotomy, and meticulous postoperative care are the definitive cornerstones for preserving hand function in these deceptive injuries.
